# Socioemotional Exchanges Between Men and Women in Heterosexual Relationships

**DOI:** 10.3389/fpsyg.2021.639302

**Published:** 2022-02-08

**Authors:** Stanley O. Gaines, Constantine Sedikides

**Affiliations:** ^1^Department of Life Sciences, Brunel University London, Uxbridge, United Kingdom; ^2^Department of Psychology, University of Southampton, Southampton, United Kingdom

**Keywords:** reward, cost, relationships, exchange, narcissism

## Abstract

We examined affection-giving, affection-denying, respect-giving, and respect-denying behaviors among men and women in heterosexual relationships. In a pilot study (*N* = 106 couples), although we had expected the latent variables of affectionate and respectful behaviors to emerge from exploratory factor analyses, we obtained the latent variables of socioemotional rewards and costs instead. In the main study (initial *N* = 182 couples), we replicated the factor patterns of socioemotional rewards and costs in confirmatory factor analyses. Moreover, we entered (final *N* = 177 couples) men’s and women’s self-reported narcissism alongside men’s and women’s socioemotional rewards and costs, as reported by partners, into a dyadic model that we tested via covariance structure analyses. Results revealed that, although men and women reciprocated rewards as well as costs (and correlations between individuals’ rewards and costs were negative), narcissism was not reflected in the patterns of reciprocity (men’s and women’s narcissism were positively related.) We discuss implications for studies of relationship processes as two-person group dynamics.

## Introduction

In an early review of the literature on close relationships, Berscheid (1985) noted that many theories within the field owe an intellectual debt to Skinner’s (1938) operant reinforcement theory regarding the presumed importance of rewards and costs to individuals’ maintenance vs. termination of relationships. Although the term “social exchange theories” often is invoked, such a term fails to capture the nuances that distinguish equity, exchange, and interdependence theories from each other (see also Berscheid and Reis, 1998). For example, Foa and Foa’s (1974) *resource exchange theory* (which posits that partners’ give-and-take of affection and respect is a hallmark of close relationships) is quite specific regarding rewards vs. costs, whereas Thibaut and Kelley’s (1959) *interdependence theory* (which proposes that partners’ mutual influence on each other’s thoughts, feelings, and behavior is a defining feature of close relationships) is non-specific (Sprecher, 1998). Moreover, resource exchange theorists have published a survey to measure particular rewards vs. costs (e.g., the Role Behavior Test or RBT; Foa and Foa, 1974); whereas interdependence theorists have not published a comparable survey (notwithstanding one-off efforts by Rusbult, 1980, 1983; see also Rusbult et al., 1986).

Following its publication in *Societal Structures of the Mind* (Foa and Foa, 1974), the RBT rarely has been used within relationship science. For instance, when we conducted a search entering the terms “resource exchange,” “Role Behavior Test,” and “Foa” via PsycInfo and Academic Search Complete (September, 2021), we uncovered two articles (Gaines, 1995; Gaines and Henderson, 2004) that had employed the RBT. Unfortunately, results of factor analyses were not reported in the book by Foa and Foa, or in the articles by Gaines (although an invitation for readers to obtain such results was offered by Gaines, 1995). Thus, we cannot be sure whether the RBT measures the constructs that it was designed to measure (i.e., affection-related and respect-related behaviors as separate, yet intercorrelated, dimensions). Consequently, without a psychometrically valid survey of affection-related and respect-related behaviors, we cannot be certain whether the basic tenets of Foa and Foa’s resource exchange theory are supported by actual data on behavioral dynamics within close relationships. In the present studies, we sought to determine whether a revised version of the RBT (Gaines and Henderson, 2004) would yield affection-related and respect-related behaviors as correlated factors.

Foa and Foa’s (1974) resource exchange theory identified several commodities (i.e., *money*, *goods*, *services*, *information*) in addition to affection/love and respect/status (Clark and Reis, 1988). In fact, their theory incorporates a circular or circumplex model (Turner et al., 1971) in which the six commodities are arrayed in an equidistant order around the behavioral axes of *particularism* (Y axis) and *concreteness* (X axis), such that affection ostensibly is more exclusive and less symbolic than is respect. However, results by Brinberg and Castell (1982) cast doubt upon the presumed ordering of commodities along those axes. Also, drawing upon Fiske’s (1991) *relational models theory* (proposing that social tasks can be classified as *communal sharing*, *equality matching*, *authority ranking*, or *market pricing*) and the Foa and Foa resource exchange theory, Haslam (1995) found that giving affection and respect clearly denoted *communality* (i.e., closeness), whereas giving information and services denoted *equality-inequality* (i.e., authority) as well as communality (giving money and goods were too infrequent in pilot research to merit inclusion). Given that later Foa and Foa (1980) came to view affection and respect as most “intangible” *and* as most likely to be exchanged within close relationships, we limit our attention to these two resources.

### Overview

In a pilot study and a main study concerning heterosexual relationships, we tested the hypothesis that (1) regarding men’s and women’s behavior separately, a two-factor model (i.e., affection-related and respect-related behaviors) would fit the correlational data significantly better than would a one-factor model (i.e., undifferentiated resource-related behaviors). Furthermore, in the pilot study as well as the main study, we tested the hypothesis that (2) men and women would exchange affection-related as well as respect-related behaviors at significant levels. Finally, in the main study (but not the pilot study), we tested the hypothesis that (3) among men and women alike, *narcissism* (one of the most intensively studied individual-difference influence on individuals’ rewarding vs. costly behaviors in general, though not necessarily studied as an influence on the particular behaviors that we have emphasized; for a review, see Muise et al., 2018) would be a significant negative predictor of individuals’ affection-related and respect-related behaviors toward their partners. Given the theme of the current special section in *Frontiers in Psychology* concerning group dynamics, we shall focus upon the potential reciprocity of affection-related and respect-related behaviors among dyads or two-person groups.

## Hypotheses Concerning the Pilot Study

In a pilot study, we tested the following hypothesis regarding the construct validity of a modified RBT (Gaines and Henderson, 2004), using a sample of heterosexual dyads: For men (whose behaviors are reported by their female partners) as well as women (whose behaviors are reported by their male partners), a two-factor model (with affection and respect as the underlying factors) will yield better fit to a matrix of interitem correlations compared to a one-factor (i.e., general) model. Given that we collected data from both members of each dyad and were especially interested in covariance between scores on men’s and women’s behaviors, we examined factor patters separately for men and women (see Berscheid, 1986, regarding the desirability of collecting and analyzing data separately when partners within each dyad can be distinguished on the basis of gender or other characteristics). We conducted exploratory factor analyses (rather than confirmatory factor analyses, given that no previously published study had entered all of the RBT items into the same factor analysis; Thompson, 2004), using the PRELIS portion of LISREL 10.2 (Joreskog and Sorbom, 2019) in tests of our hypothesis. For all of the analyses that follow, details concerning input (e.g., syntax/code) and output (e.g., tables/text) are available from the first author upon request.

### Method

#### Participants

We obtained ethics approval from the Psychology Ethics Committee at the first author’s academic institution, consistent with the British Psychological Society Code of Ethics and Conduct (British Psychological Society, 2018). We relied on a convenience sample, with dyads (i.e., pairs of participants in heterosexual relationships) recruited by our research assistants via snowball sampling. Our remit to research assistants was broad: Acquaintances and non-acquaintances of theirs could be recruited via e-mail, text, social media, face-to-face interaction, and/or other means. We tested 106 heterosexual couples (106 men, 106 women), all volunteers. Men’s mean age was 27.34 years (*SD* = 11.49 years), and women’s mean age was 25.32 years (*SD* = 11.12 years). A majority of participants classified themselves as White/European-descent (for men: 61.9% White/European-descent, 13.6% Asian-descent, 8.5% Black/African-descent, 4.2% “Mixed,” 1.7% “Other,” 10.2% unreported; for women: 62.7% White/European-descent, 16.1% Asian-descent, 10.2% Black/African-descent, 0.8% “Mixed,” 10.2% unreported; further details regarding ethnic group membership of participants are available from the first author upon request, consistent with the more specific categories that are recognized by the UK Office for National Statistics (2012). A plurality of participants did not specify their educational status, checking the box “other” (for men, 5.1% first-year undergraduate, 11.0% second-year undergraduate, 9.3% third-year undergraduate, 6.8% fourth-year undergraduate, 48.3% “other,” 19.5% unreported; for women, 11.9% first-year undergraduate, 25.4% second-year undergraduate, 4.2% third-year undergraduate, 4.2% fourth-year undergraduate, 36.4% “other,” 17.8% unreported). Lastly, in terms of occupation, a plurality of participants listed themselves as full-time students (for men, 22.0% professional/managerial, 22.0% clerical/sales/skilled labor, 8.5% services/unskilled labor, 0.8% homemaker, 30.5% full-time student, 5.1% retired/unemployed/job-seeking, 11% unreported; for women, 11.9% professional/managerial, 10.2% clerical/sales/skilled labor, 5.1% services/unskilled labor, 8.5% homemaker, 46.6% full-time student, 7.6% retired/unemployed/jobseeking, 10.2% unreported).

#### Materials and Procedure

Participants completed a 12-item, modified version of the RBT (Gaines and Henderson, 2004) along with additional social-psychological and individual-difference variables that were pertinent to another project. The modified RBT had been developed by Gaines et al. (1999) to remove “double-barreled” questions (whereby participants are required to provide one response to two mini-questions that are joined together linguistically but are distinct conceptually; Olson, 2008) prevalent in Foa and Foa’s (1974) original RBT. The modified RBT was designed to measure the relative frequency with which individuals reported that their partners had given them affection (3 items), denied them affection (3 items), given them respect (3 items), and denied them respect (3 items) during the two weeks prior to taking part in the study. Sample items include: “My partner has expressed warmth toward me” (affection-giving); “My partner has withheld love from me” (affection-denying); “My partner has encouraged my personal growth” (respect-giving); and “My partner has treated me with disrespect” (respect-denying) (1 = *almost never*, 5 = *almost always*).

### Results and Discussion

As Thompson (2004) pointed out, even if researchers hold *a priori* expectations regarding factor patterns, the process of establishing construct validity for a given survey ideally should include exploratory factor analyses on data from an initial sample, followed by confirmatory factor analyses on data from a subsequent sample (see also Tabachnick and Fidell, 2009). However, such a step-by-step process is not evident from published articles concerning Foa and Foa’s (1974) original RBT (e.g., Gaines, 1995) or a revised version of the RBT (e.g., Gaines and Henderson, 2004). Therefore, in the pilot study, we prioritized conducting exploratory factor analyses upon data from the revised RBT. Kaiser’s (1970) “little jiffy” method (whereby each factor with an eigenvalue of 1.00 of greater is retained) was applied automatically by PRELIS in an effort to identify the optimal number of factors.

#### Men’s Interpersonal Behavior (as Reported by Women)

To determine the optimal number of factors for the items that measured men’s interpersonal behavior (as reported by their female partners), we conducted an exploratory factor analysis with maximum likelihood estimation. Initially, we did not request a solution with a particular number of factors; inspection of the accompanying decision table (shown in Table 1) revealed that PRELIS had attempted to extract as many as three factors. However, inspection of Varimax-rotated and Promax-rotated matrices of loadings for a three-factor solution yielded uninterpretable results (i.e., Heywood cases or instances in which communalities for one or more items exceeded 1.00; Thompson, 2004). Clearly, the factor extraction procedure for men’s behavior items was insufficient to produce a stable solution in the absence of an explicit specification of a lower number of factors (a not-infrequent problem in exploratory factor analysis; Tabachnick and Fidell, 2009). Subsequently, we re-ran the exploratory factor analysis, requesting a two-factor solution; unexpectedly, the resulting matrix of loadings for the Promax-rotated solution (shown in Table 2, taking into account the correlation between the two factors, which was −0.50) revealed that Factor 1 consisted of *rewards* (i.e., affection-giving and respect-giving behaviors), whereas Factor 2 consisted of *costs* (i.e., affection-denying and respect-denying behaviors). Unlike the Promax-rotated solution, the matrix of loadings for the Varimax-rotated solution (shown in Table 2, without taking into account the correlation between the two factors) did not yield a “clean” separation of items onto particular factors (i.e., for two items, absolute values for loadings were 0.32 or higher on both factors; see Tabachnick and Fidell, 2009, regarding recommended cutoff points for factor loadings).

**TABLE 1:** Decision tables for number of interpersonal behavior factors in the pilot study (*N* = 106 couples).^a^

Chi-model	MLDF	Square	*p*	RMSEA	GFI	AGFI	*df*	EP
**Men**’**s interpersonal behavior (reported by women)**								
1-factor	–	461.96	<0.01	0.27	–	–	54	–
2-factor	–	223.29	<0.01	0.20	–	–	43	–
**Women**’**s interpersonal behavior (reported by men)**								
1-factor	–	406.25	<0.01	0.25	–	–	54	–
2-factor	–	178.84	<0.01	0.17	–	–	43	–

*^a^MLDF, Maximum likelihood discrepancy function; RMSEA, root mean square error of approximation; GFI, goodness-of-fit index; EP, number of parameters to be estimated. Values for MLDF, GFI, AGFI, and EP are not provided by the PRELIS portion of LISREL 10 (Joreskog and Sorbom, 2019), which is relevant to exploratory factor analyses.*

**TABLE 2:** Loadings for men’s and women’s interpersonal behavior items in the pilot study (*N* = 106 couples)^a^.

	Varimax rotation		Promax rotation	
Item	Rewards	Costs	Rewards	Costs
**Men**’**s interpersonal behavior (reported by women)**				
1	0.85	–0.12	0.91	0.11
2	0.89	–0.11	0.96	0.14
3	0.75	–0.15	0.79	0.05
4	–0.26	0.69	–0.08	0.69
5	–0.34	0.72	–0.16	0.71
6	–0.23	0.77	–0.02	0.8
7	0.64	–0.32	0.62	–0.17
8	0.53	–0.36	0.48	–0.24
9	0.59	–0.28	0.57	–0.14
10	–0.1	0.91	0.17	0.99
11	–0.2	0.85	0.04	0.9
12	–0.22	0.89	0.02	0.93
**Women**’**s interpersonal behavior (reported by men)**				
1	0.83	–0.12	0.89	0.1
2	0.86	–0.25	0.88	–0.03
3	0.78	–0.2	0.81	0
4	–0.24	0.65	–0.05	0.66
5	–0.36	0.79	–0.14	0.78
6	–0.38	0.74	–0.19	0.72
7	0.54	–0.29	0.51	–0.18
8	0.68	–0.27	0.67	–0.11
9	0.78	–0.2	0.8	–0.01
10	–0.06	0.76	0.18	0.83
11	–0.15	0.78	0.08	0.83
12	0.83	–0.12	0.89	0.1

*^a^1. My partner has expressed warmth toward me.*

*2. My partner has shown a sense of belonging toward me.*

*3. My partner has shown enjoyment toward me.*

*4. My partner has withheld love from me.*

*5. My partner has failed to show tenderness toward me.*

*6. My partner has shown lack of closeness toward me.*

*7. My partner has encouraged my personal growth.*

*8. My partner has recognized my personal accomplishments.*

*9. My partner has made me feel like an important person.*

*10. My partner has treated me with disrespect.*

*11. My partner has been unappreciative of me as a unique person.*

*12. My partner has failed to show confidence in my abilities.*

In absolute terms, neither the one-factor solution nor the two-factor solution provided satisfactory fit to the data [i.e., chi-squares *p* < 0.01, combined with root mean square errors of approximation (RMSEA) greater than 0.10—Schumacker and Lomax, 2016]. Nevertheless, results of the exploratory factor analyses for men’s interpersonal behavior (reported by women) indicated that a two-factor solution provided better fit than a one-factor solution was supported (reduction in chi-square = 238.67, reduction in degrees of freedom = 11, *p* < 0.01). Contrary to hypotheses, the content of the two-factor solution represented rewards and costs as anticipated by the original version of Thibaut and Kelley’s (1959) interdependence theory—*not* affection-related and respect-related behaviors as anticipated by Foa and Foa’s (1974) resource exchange theory, despite the origins of the modified RBT (Gaines and Henderson, 2004) in that theory.

#### Women’s Interpersonal Behavior (as Reported by Men)

Subsequently, with regard to women’s interpersonal behavior (as reported by their male partners), we conducted an exploratory factor analysis with maximum likelihood estimation. As was the case for men’s interpersonal behavior (reported by women), we did not request a particular number of factors in our initial exploratory factor analysis of women’s interpersonal behavior. However, unlike the initial exploratory factor analysis for men’s interpersonal behavior, the initial exploratory factor analysis for women’s interpersonal behavior produced a decision table (Table 1) with no more than two factors (and without any problematic Heywood cases). We did not need to specify the number of factors for women’s behavior items (although, in principle, we could have used the results for men’s behavior as justification for setting the number of factors at two for women’s behavior). In any event, as indicated by the Promax-rotated factor loadings (Table 2), we replicated the unanticipated factors of socioemotional rewards and costs that we had obtained for men’s interpersonal behavior (the correlation between women’s rewards and costs, −0.50, was identical to the correlation that we found between men’s rewards and costs). Finally, similar to what we observed for men’s interpersonal behavior, results of the Varimax-rotated solution for women’s interpersonal behavior (Table 2) did not produce a clean set of loadings on particular factors.

In absolute terms, the two-factor solution did not provide satisfactory fit to the data (i.e., significant chi-square combined with RMSEA greater than 0.10; Table 2). However, as was true for men’s interpersonal behavior (reported by women), results of the exploratory factor analyses for women’s interpersonal behavior (reported by men) indicated that a two-factor solution provided better fit than a one-factor solution was supported (reduction in chi-square = 227.41, reduction in degrees of freedom = 11, *p* < 0.01). Given the lack of absolute goodness-of-fit for the two-factor solution for women’s as well as men’s interpersonal behavior—in spite of the fact that the two-factor solution proved to be optimal for women’s as well as men’s interpersonal behavior—we wondered whether built-in limitations of exploratory factor analyses in general (requiring the calculation of loadings for all items on all factors, inability to incorporate inter-factor correlations into models) prevented us from obtaining two-factor solutions with satisfactory goodness-of-fit to the correlational data (Thompson, 2004).

#### Internal Consistency Coefficients and Correlations Involving Men’s and Women’s Behavioral Subscales

Results of reliability analyses indicated that the scales measuring men’s rewards, men’s costs, women’s rewards, and women’s costs were internally consistent, with internal consistency coefficients exceeding 0.80 for all four scales (Cronbach’s alphas = 0.89 for men’s rewards, 0.94 for men’s costs, 0.90 for women’s rewards, and 0.91 for women’s costs). In addition, all of the correlations among scores on the four behavior scales (shown in Table 3) were significant (*p*s < 0.01), with the only positive correlations occurring between men’s and women’s rewards, and men’s and women’s costs. Notwithstanding the unexpected patterns of “giving” and “denying” items loading onto separate factors, the reconfigured behavior scales were low in measurement error and were intercorrelated and in directions that align with conceptualizations of rewards and costs in the original version of interdependence theory (Thibaut and Kelley, 1959).

**TABLE 3:** Correlations among total scores on interpersonal behavior subscales in the pilot study (*N* = 106 couples).^a^

	Correlations			
Var.	1	2	3	4
1	1			
2	–0.47	1		
3	0.43	–0.31	1	
4	–0.35	0.65	–0.51	1

*^a^All correlations are significant (ps < 0.001 or below).*

*1 = Men’s socioemotional rewards (reported by women).*

*2 = Men’s socioemotional costs (reported by women).*

*3 = Women’s socioemotional rewards (reported by men).*

*4 = Women’s socioemotional costs (reported by men).*

Although we did not propose any hypotheses concerning mean differences between men’s and women’s socioemotional rewards or costs, we supplemented correlational analyses with paired-sample *t*-tests via SPSS 26.0 (IBM, 2019). Results of paired-sample *t*-tests indicated that men and women did not differ on rewards or costs. Details are available from the first author upon request.

#### Transition From Pilot Study to Main Study: (Re)Casting the Role Behavior Test as a Measure of Socioemotional Rewards and Costs That May Be Exchanged

Earlier in this article, we alluded to Haslam’s (1995) results concerning affection-giving and respect-giving behavior items as loading on a single, communality/closeness factor (apparently following a principal axis factor analysis, although Haslam did not specify the type of exploratory factor analysis; Thompson, 2004). Just as Haslam and Fiske (1999) subsequently re-evaluated core assumptions of Foa and Foa’s (1974) resource exchange theory concerning the usefulness of the affection-respect distinction in light of Haslam’s (1995) earlier results, so too did we begin to question key assumptions of that theory concerning the utility of the affection-respect distinction when reflecting upon our own pilot study results. However, unlike Haslam and Fiske (1999), we did not discard the RBT items in favor of alternative items (e.g., items that were designed to be compatible with the relational models theory of Fiske, 1991). Instead, influenced by Kelley et al.’s (1983/2002) argument that interdependence is a defining feature of close relationships, we *re-interpreted* the RBT items from the standpoint of Thibaut and Kelley’s (1959) interdependence theory (initially revised by Kelley and Thibaut, 1978, and subsequently refined by Kelley, 1979).

Given the results that we obtained for the modified RBT (Gaines and Henderson, 2004), we will refer to affection-giving, respect-giving, affection-denying, and respect-denying behaviors henceforth as *socioemotional rewards and costs* (following Lawler and Thye, 1999). In addition to shifting our terminology, we shall shift our conceptual focus from Foa and Foa’s (1974) resource exchange theory to Thibaut and Kelley’s (1959) interdependence theory via Jerry Wiggins’s (2003/2006) *interpersonal circumplex theory of personality and social behavior* (a theory that straddles the traditional boundary between personality psychology and social psychology). The following quote from Wiggins (1979) (p. 398), citing Foa and Foa’s theory, captures our logic concisely: “…[I]nterpersonal events may be defined as *dyadic interactions that have relatively clear-cut social (status) and emotional (love) consequences for both participants (self and other)*” (emphasis in original). In turn, Kelley (1997) cited Wiggins’s theory, suggesting that individuals will be inclined to remain in relationships to the extent that individuals are dependent upon their partners for status/respect and love/affection (though the level of dependence may not be mutual; Reis et al., 2002).

To what extent are socioemotional rewards and costs *exchanged* within heterosexual relationships? Drawing upon an early version of Wiggins’s interpersonal circumplex theory of personality and social behavior (Wiggins, 1979), Kelley (1983) contended that genuine reciprocity is most likely to occur in relationships within which individuals and their partners share the perception that their relationships are equal (see also Wish et al., 1976). Under such circumstances, mutual dependence will be the behavioral norm (see also Kelley and Thibaut, 1978). Although Thibaut and Kelley’s (1959) original version of interdependence theory did not prioritize the cognitive aspects of mutual dependence (Kelley, 1997), successive revisions of interdependence theory (Kelley and Thibaut, 1978; Kelley, 1979) acknowledged the role that individuals’ consciously experienced, prosocial goals may play in fostering reciprocity of socioemotional rewards and costs within close relationships (including, but not limited to, heterosexual relationships; Holmes, 2000). We hasten to add that (1) individuals may pursue self-interested (rather than prosocial) goals; and (2) unilateral (rather than mutual) dependence may emerge as an alternative behavioral norm, especially in heterosexual romantic relationships (often favoring men over women; Holmes, 2002).

#### Adding Narcissism as a Potential Predictor of Socioemotional Rewards and Costs That May Be Exchanged in the Main Study

Wiggins’s (1979) initial version of interpersonal circumplex theory emphasized *traits* (i.e., individuals’ answer to the question, “What are you like?”) as personality influences on socioemotional rewards and costs. However, Wiggins (1991) subsequently proposed an *interpersonal circumplex theory of personality and social behavior* that identified Bakan’s (1966) prior dichotomy between *agency* (an intrapersonal orientation) and *communion* (an interpersonal orientation) as two overarching modalities of “being-in-the-world” that characterize the human experience (see also Wiggins, 2003/2006). Although Wiggins emphasized the agentic trait of *dominance* and the communal trait of *nurturance* (Wiggins and Broughton, 1991), Wiggins’s (1997) expanded theory also includes *motives* (i.e., individuals’ answer to the question, “What drives you to behave as you do?”—noting that individuals are not necessarily aware of their motives), particularly the agentic motive of *power* and the communal motive of *intimacy*. Moreover, Wiggins’s expanded theory arguably encompasses *attitudes* (i.e., individuals’ answer to the question, “How do you evaluate that object?”), specifically the agentic attitude of *attachment anxiety* (reverse-scored) and the communal attitude of *attachment avoidance* (reverse-scored; see also Bartholomew, 1990).

Taking on board various aspects of personality that interpersonal circumplex theorists (following Wiggins, 1991) have identified, a most promising individual-difference influence on socioemotional rewards and costs may be a construct that is not prominent within Wiggins’s theory. This construct is, *narcissism*, a trait that reflects both egocentric exceptionalism (beliefs on one’s superiority, specialness, importance, and entitled) and social selfishness (looking down on others unempathetically and even antipathetically; Sedikides, 2021). As several authors pointed out (Sedikides et al., 2004; Krizan and Herlache, 2018; Thomaes et al., 2018), narcissism—grandiose narcissism, in particular—is consistently aligned with constructs that occupy the high agency/low communion position within circumplex models of personality, from the blended interpersonal trait of arrogant-calculating to the blended interpersonal attitude of dismissing-avoidant. In turn, high agency/low communion combinations may predispose individuals to bestow socioemotional rewards upon *themselves*, yet inflict socioemotional costs upon their partners (Hopwood and Waugh, 2020).

Does it necessarily follow that narcissism will be associated negatively with individuals’ bestowal of benefits toward their *partners*, and positively with individuals’ inflicting of costs upon their partners? Work by Campbell et al.’s (2000, 2002) work concerning the likely consequences of narcissism for individuals’ behavior within close relationships is consistent with such a conclusion, although these authors did not explicitly refer to Wiggins’s (1991) revised interpersonal circumplex theory or Kelley and Thibaut (1978) revised interdependence theory. Consistent with that conclusion is also a large literature on narcissism in relationships (Seidman, 2016; Gewirtz-Meydan, 2017; Brewer et al., 2020; for reviews, see Brunell and Campbell, 2011; Sedikides, 2021). Moreover, although a synthesis of interpersonal circumplex theory and interdependence theory (Gaines, 2016/2018) likewise would support such a conclusion, the literature on interdependence processes has been more likely to address the related construct of *self-esteem* (denoting individuals’ more realistic attitude toward themselves; Brummelman et al., 2016, 2018) as a positive influence on individuals’ bestowal of rewards—and a negative influence on individuals’ inflicting of costs—toward partners (Machia et al., 2020). Thus, our hypotheses concerning the role of narcissism on individuals’ socioemotional rewards and costs in heterosexual relationships are tentative.

In the preceding two paragraphs, we implicitly drew upon Sullivan’s (1953) *interpersonal theory of personality* (which proposes that individual differences outside the domain of intelligence are best understood as enacted within the context of individuals’ relationships with important others; see also Sullivan, 1954) in referring to narcissism and self-esteem. Given that echoes of Sullivan’s theory reverberate through the initial versions of Thibaut and Kelley’s (1959) interdependence theory, Wiggins’s (1997) interpersonal circumplex theory, and even Foa and Foa’s (1974) resource exchange theory, Sullivan’s theory serves as a conceptual framework that allows us to integrate seemingly disparate strands of research from personality psychology and relationship science (Gaines, 2016/2018). Especially relevant to our main study is Sullivan’s contention that, unlike realistically informed self-esteem (which incorporates “bad-me” as well as aspects of personality), narcissism reflects individuals’ misinterpretation of “bad-me” aspects of personality as “not-me” (Ewen, 1998).

## Hypotheses Concerning the Main Study

In our main study, we tested the following revised hypothesis concerning the construct validity of the revised RBT (Gaines and Henderson, 2004), using a separate sample of heterosexual dyads: (1) For men (whose behaviors are reported by their female partners) as well as women (whose behaviors are reported by their male partners), a two-factor model (with socioemotional rewards and costs as the underlying factors) will yield better fit to a matrix of interitem correlations when the factors are allowed to be correlated, rather than uncorrelated. Additionally, we tested the following set of hypotheses regarding the criterion-related validity of the modified RBT: (2) (a) men and women will reciprocate socioemotional rewards; (b) men and women will reciprocate socioemotional costs; (c) among men and women, socioemotional rewards and costs will be negatively correlated; (d) among men and women, narcissism will be a negative predictor of socioemotional rewards; also among men and women, narcissism will be a positive predictor of socioemotional costs. Building on the results from our pilot study, we conducted confirmatory factor analyses and covariance structure analyses (Kline, 2016), using the main portion of LISREL 10.2 (Joreskog and Sorbom, 2019). Unlike exploratory factor analysis, factor rotation is a non-issue in confirmatory factor analysis (Thompson, 2004). Thus, we do not distinguish between unrotated and rotated factor solutions in the main study.

### Method

#### Participants

The research protocol was similar to that (including ethics approval and participant recruitment) of the pilot study, consistent with the British Psychological Society Code of Ethics and Conduct (British Psychological Society, 2018). We tested 182 heterosexual couples (182 men, 182 women). Mean age for men was 34.90 years (*SD* = 13.67 years) and for women 33.37 years (*SD* = 13.36 years). Approximately half of participants classified themselves as White/European-descent (for men: 49.0% White/European-descent, 22.5% Asian-descent, 21.9% Black/African-descent, 5.5% “Mixed,” 0.5% “Other,” 0.5% unreported; for women: 49.9% White/European-descent, 29.1% Asian-descent, 14.2% Black/African-descent, 3.8% “Mixed,” 2.7% unreported; further details regarding ethnic group membership of participants are available from the first author upon request. A plurality of participants checked the box “other” for educational status (for men: 4.4% first-year undergraduate, 8.2% second-year undergraduate, 7.7% third-year undergraduate, 4.4% fourth-year undergraduate, 36.6% “other,” 38.8% unreported; for women, 4.9% first-year undergraduate, 13.7% second-year undergraduate, 8.7% third-year undergraduate, 4.9% fourth-year undergraduate, 33.3% “other,” 34.4% unreported). Lastly, in terms of occupation, a plurality of men listed themselves as professional/managerial, whereas a plurality of women listed themselves as full-time students (for men, 36.6% professional/managerial, 21.9% clerical/sales/skilled labor, 9.3% services/unskilled labor, 0.5% homemaker, 23.5% full-time student, 7.7% retired/unemployed/job-seeking, 0.5% unreported; for women, 19.7% professional/managerial, 15.8% clerical/sales/skilled labor, 7.7% services/unskilled labor, 14.8% homemaker, 32.8% full-time student, 8.7% retired/unemployed/jobseeking, 0.5% unreported).

#### Materials and Procedure

##### Socioemotional Rewards and Costs

Participants completed the aforementioned, modified 12-item version of the RBT (Gaines and Henderson, 2004).

##### Narcissism

Participants filled out the 40-item Narcissistic Personality Inventory (NPI; Raskin and Hall), a validated and widely used measure of grandiose narcissism (Emmons, 1984; Prifitera and Ryan, 1984; Watson et al., 1984; Raskin and Terry, 1988; for a review, see Miller and Campbell, 2011). Each item consists of a pair of statements—one narcissistic, one non-narcissistic. The number of narcissistic statements that participants endorse is their narcissism score (Cronbach’s alphas = 0.90 for men and 0.88 for women). Although Rosenthal and Hooley (2010) concluded that the NPI includes several items that measure self-esteem instead of narcissism, Miller et al. (2011) did not find evidence of such a self-esteem/narcissism confounding pattern within the NPI.

### Results and Discussion

Consistent with Thompson’s (2004) aforementioned recommendations, having obtained socioemotional rewards and costs as the two dimensions that are measured by the revised RBT (Gaines and Henderson, 2004) via exploratory factor analyses in the pilot study, we were in a position to try and replicate that pattern of latent variables via confirmatory factor analyses in the main study (see also Tabachnick and Fidell, 2009). As was the case for the polit study, we conducted separate analyses for men’s socioemotional rewards and costs (as reported by women), followed by analyses for women’s socioemotional rewards and costs (as reported by men) in the main study. Details concerning all input and output information are available from the first author upon request.

#### Men’s Socioemotional Rewards and Costs (as Reported by Women)

To test our hypothesis regarding the two-factor pattern and exclusion vs. inclusion of an interfactor correlation for men’s socioemotional rewards and costs (as reported by their female partners), we conducted a pair of confirmatory factor analyses. We made the following specifications: (1) In the theta epsilon (TE, or measurement error) matrix, we freed uncorrelated measurement error terms associated with the 12 modified RBT items (Gaines and Henderson, 2004), but constrained them to be equal to each other (all correlated measurement error terms were fixed at 0.00); (2) in the lambda Y (LY, or latent-observed variable) matrix, we freed loadings for the three affection-giving items and three respect-giving items on Factor 1 (rewards), whereas we freed loadings for the three affection-denying items and three respect-denying items on Factor 2 (costs), with all other loadings fixed at 0.00; and (3) in the psi (PS, or variance-covariance) matrix, we freed the error variance terms for the reward and cost factors at 1.00 (for details regarding LISREL syntax, see Mels, 2020; Scientific Software International, 2020). We estimated all freed parameters via the maximum likelihood method, with the ridge option and ridge constant, given the problems with communalities that we had encountered when we conducted exploratory factor analyses of the RBT in the pilot study.

In the initial two-factor model, the correlation between men’s reward and cost factors was fixed at 0.00. Results of a confirmatory factor analysis indicated that (as expected) the initial model did not yield satisfactory fit to the interitem correlation data (see goodness-of-fit statistics in Table 4). Not only was the chi-square significant (*p* < 0.01), but the maximum likelihood discrepancy function was unacceptably high (and the unadjusted as well as adjusted goodness-of-fit indices were lower than optimal; Schumacker and Lomax, 2016). Given that the orthogonal version of the two-factor model did not provide adequate fit to the data, we will not interpret factor loadings from this particular analysis.

**TABLE 4:** Decision tables for uncorrelated vs. correlated socioemotional reward and cost factors in the main study (initial *N* = 182 couples).^a^

Chi-model	MLDF	Square	*P*	RMSEA	GFI	AGFI	*df*	*df*
**Men**’**s socioemotional rewards and costs (reported by women)**								
2 uncor. factors	0.19	99.38	<0.01	0.05	0.92	0.91	65	13
2 cor. factors	0.00	33.12	*NS*	0.00	0.97	0.96	64	14
**Women**’**s socioemotional rewards and costs (reported by men)**								
2 uncor. factors	0.16	94.11	<0.01	0.05	0.93	0.91	65	13
2 cor. factors	0.00	34.37	*NS*	0.00	0.97	0.96	64	14

*^a^MLDF, Maximum likelihood discrepancy function; RMSEA, root mean square error of approximation; GFI, goodness-of-fit index; EP, number of parameters to be estimated.*

By contrast, in the final two-factor model, we freed the correlation between men’s reward and cost factors. Results of a confirmatory factor analysis indicated that (as expected) the final model yielded satisfactory fit to the interitem correlation data for men’s rewards and costs (see Table 5 regarding goodness-of-fit statistics). Not only was the chi-square non-significant, but the maximum likelihood discrepancy function was zero (and the unadjusted as well as adjusted goodness-of-fit indices were above 0.95). Also, the reduction in chi-square from the initial to final model (66.26) was significant (reduction in degrees of freedom = 1; resulting *p* < 0.01). Furthermore, all non-zero factor loadings (Table 6) were significant (*p*s < 0.01) and positive, exceeding 0.50 in value. Finally, the correlation between men’s reward and cost factors was negative (*r* = −0.80, *p* < 0.01). The very high correlation reflected that (unlike exploratory factor analyses) confirmatory factor analyses allow researchers to control statistically for measurement error (for an in-depth examination of confirmatory factor analysis, see Brown, 2015; cf. Onde and Alvarado, 2018).

**TABLE 5:** Correlations among total scores on narcissism scale and socioemotional reward and cost subscales in the main study (final *N* = 177 couples).^a^

	Correlations						
Var.	1	2	3	4	5	6
1	1					
2	0.01	1				
3	0.07	–0.7	1			
4	0.04	0.49	–0.3	1		
5	0.07	–0.47	0.44	–0.65	1	
6	0.28	0.07	0.09	0.05	0.19	1

*^a^All correlations greater than 0.15 in absolute value are significant (ps < 0.050 or below).*

*1 = Men’s self-reported narcissism.*

*2 = Men’s socioemotional rewards (reported by women).*

*3 = Men’s socioemotional costs (reported by women).*

*4 = Women’s socioemotional rewards (reported by men).*

*5 = Women’s socioemotional costs (reported by men).*

*6 = Women’s self-reported narcissism.*

**TABLE 6:** Loadings for men’s and women’s socioemotional reward and cost items in the main study (initial *N* = 182 couples).^a^

Item	Rewards	Costs
**Men**’**s socioemotional rewards and costs (reported by women)**		
1	0.77	0
2	0.79	0
3	0.77	0
4	0	0.71
5	0	0.85
6	0	0.83
7	0.6	0
8	0.65	0
9	0.74	0
10	0	0.74
11	0	0.7
12	0	0.64
**Women**’**s socioemotional rewards and costs (reported by men)**		
1	0.68	0
2	0.73	0
3	0.74	0
4	0	0.57
5	0	0.79
6	0	0.75
7	0.61	0
8	0.73	0
9	0.75	0
10	0	0.64
11	0	0.71
12	0	0.72

*^a^1. My partner has expressed warmth toward me.*

*2. My partner has shown a sense of belonging toward me.*

*3. My partner has shown enjoyment toward me.*

*4. My partner has withheld love from me.*

*5. My partner has failed to show tenderness toward me.*

*6. My partner has shown lack of closeness toward me.*

*7. My partner has encouraged my personal growth.*

*8. My partner has recognized my personal accomplishments.*

*9. My partner has made me feel like an important person.*

*10. My partner has treated me with disrespect.*

*11. My partner has been unappreciative of me as a unique person.*

*12. My partner has failed to show confidence in my abilities.*

#### Women’s Socioemotional Rewards and Costs (as Reported by Men)

We conducted the same pair of confirmatory factor analyses on the RBT data for women’s socioemotional rewards and costs (as reported by men) that we had carried out on the RBT data for men’s socioemotional rewards and costs (i.e., two-factor model with uncorrelated factors, followed by two-factor model with correlated factors). Once again, we used LISREL 10.2 (Joreskog and Sorbom, 2019) to run the analyses, incorporating maximum likelihood estimation, ridge option, and ridge constant.

Results of a confirmatory factor analysis indicated that, as expected, the initial model (i.e., two uncorrelated factors) did not yield satisfactory fit to the interitem correlation data for women’s rewards and costs (goodness-of-fit statistics are presented in Table 4). As was the case for men’s rewards and costs, not only was the chi-square significant (*p* < 0.01), but the maximum likelihood discrepancy function was unacceptably high (and the unadjusted as well as adjusted goodness-of-fit indices were lower than optimal). Given that the orthogonal version of the two-factor model did not provide adequate fit to the data, we will not interpret factor loadings from this analysis.

Subsequently, results of a confirmatory factor analysis indicated that (as expected) the final model yielded satisfactory fit to the interitem correlation data for women’s rewards and costs (see Table 4 regarding goodness-of-fit statistics). As was true of the final model for men’s rewards and costs, not only was the chi-square non-significant, but the maximum likelihood discrepancy function was zero (and the unadjusted as well as adjusted goodness-of-fit indices were above 0.95). Also, the reduction in chi-square from the initial to final model (59.74) was significant (reduction in degrees of freedom = 1; resulting *p* < 0.01). Furthermore, all non-zero factor loadings (shown in Table 6) were significant (*p*s < 0.01) and positive, exceeding 0.50 in value. Finally, the correlation between women’s reward and cost factors was negative (*r* = −0.80, *p* < 0.01)—again, due to the ability of confirmatory factor analyses to control statistically for measurement error (Brown, 2015; cf. Onde and Alvarado, 2018).

#### Internal Consistency Coefficients and Correlations Involving Men’s and Women’s Socioemotional Rewards and Costs

As in the pilot study, results of reliability analyses indicated that the scales measuring men’s rewards, men’s costs, women’s rewards, and women’s costs in the main study were internally consistent, with internal consistency coefficients exceeding 0.80 (Cronbach’s alphas = 0.86 for men’s rewards, 0.89 for men’s costs, 0.85 for women’s rewards, and 0.85 for women’s costs)—somewhat higher than we had obtained for the RBT subscales in the pilot study. Also, all of the correlations among scores on the four behavior scales were significant (*p*s < 0.01), with the only positive correlations occurring (1) between men’s and women’s rewards, and (2) between men’s and women’s costs. In sum, men’s and women’s socioemotional reward and cost scales were low in measurement error, and were intercorrelated in directions congruent with interdependence theory (Thibaut and Kelley, 1959).

Having replicated our pilot study results for internal consistencies and correlations among the RBT scales measuring men’s rewards, men’s costs, women’s rewards, and women’s costs, we concluded that we could incorporate the dynamics of men’s and women’s reciprocity of rewards, men’s and women’s reciprocity of costs, men’s positive correlation between their bestowal of rewards and costs, and women’s positive correlation between their bestowal of rewards and costs into the core of a covariance structure model concerning male-female interactions in the situational context of heterosexual relationships. Moreover, having measured men’s and women’s narcissism in the main study, we were in a position to add individual-difference variables to the model: men’s narcissism as a predictor of men’s rewards (negative effect) and costs (positive effect), as well as women’s narcissism as a predictor of women’s rewards (negative effect) and costs (positive effect). Therefore, we conducted covariance structure analyses to test the model as a whole, along with the correlations and beta coefficients that we expected.

Although we did not propose hypotheses concerning mean gender differences in narcissism, socioemotional rewards, or socioemotional costs, we supplemented our correlation analysis with a series of paired-sample *t*-tests via SPSS 26.0 (IBM, 2019). Results indicated that men scored higher than women on narcissism (*p* < 0.01), although men and women did not differ on socioemotional rewards or costs. Details regarding the paired-sample *t*-tests are available from the first author upon request.

#### Men’s and Women’s Narcissism, Socioemotional Rewards, and Socioemotional Costs: Testing the Covariance Structure Model

Among five couples, men and/or women did not respond to one or more NPI items, leaving us with a slightly reduced sample of 177 couples for testing the covariance structure model. We present in Table 5 the matrix of correlations among total scores for (1) men’s narcissism, (2) men’s rewards (as reported by women), (3) men’s costs (as reported by women), (4) women’s rewards (as reported by men), (5) women’s costs (as reported by men), and (6) women’s narcissism. We entered this matrix into two covariance structure analyses with maximum likelihood estimation, ridge option, and ridge constant. Although we had planned to conduct only one covariance structure analysis, results of that initial analysis (as will become evident shortly) indicated that we should account for an unexpected, positive correlation between men’s and women’s narcissism in a subsequent analysis (keeping in mind that such an addition technically represents a shift from a confirmatory mode to an exploratory mode of analysis; Kline, 2016).

**FIGURE 1:**
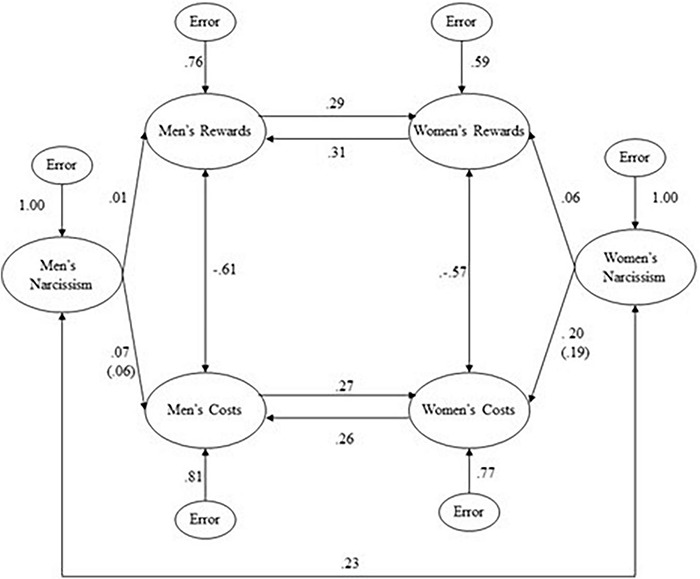
Covariance structure model of men’s and women’s narcissism, rewards, and costs (final *N* = 177 couples). All beta coefficients and correlations with absolute values greater than 0.25 are significant (*ps* < 0.05 or below).

In an initial covariance structure analysis, we specified the following parameters: (1) in the TE matrix, we freed all uncorrelated measurement error terms for the full scales but constrained the error terms to be equal (all correlated measurement error terms were fixed at 0.00); (2) in the LY matrix, we fixed loadings for all full scales on their respective factors at 1.00; (3) in the BE (i.e., beta coefficient) matrix, we freed unidirectional paths from men’s narcissism to men’s rewards and costs, freed unidirectional paths from women’s narcissism to women’s rewards and costs, freed bidirectional (i.e., reciprocal) paths between men’s and women’s rewards, we freed bidirectional paths between men’s and women’s costs; and (4) in the PS matrix, we freed unexplained variance terms for men’s rewards, men’s costs, women’s rewards, and women’s costs; and we freed correlations between men’s rewards and costs, and between women’s rewards and costs (we fixed unexplained variance paths for men’s narcissism and women’s narcissism at 1.00). As shown in Table 7, the goodness-of-fit statistics indicate that the initial model provided satisfactory fit to the correlational data (chi-square was non-significant; all other goodness-of-fit statistics were acceptable). Further inspection of the estimated parameters (Figure 1) revealed that, although all of the beta coefficients and correlations within the core of the covariance structure model were significant and in the expected direction (*p*s < 0.01), the paths from men’s and women’s narcissism to the reward and cost variables were non-significant. The only path that showed promise in terms of magnitude was the positive path from women’s narcissism to women’s costs; and the standard error for that path was so large that the resulting significance level was above 0.10 (Kline, 2016).

**TABLE 7:** Decision tables for covariance structure model, uncorrelated vs. correlated scores for men’s and women’s narcissism, main study (final *N* = 177 couples).^a^

Chi-model	MLDF	Square	*p*	RMSEA	GFI	AGFI	*df*	EP
Uncor. narc.	0.00	5.22	*NS*	0.00	1.00	0.99	8	13
Cor. narc.	0.00	1.59	*NS*	0.00	1.00	1.01	7	14

*^a^MLDF, Maximum likelihood discrepancy function; RMSEA, root mean square error of approximation; GFI, goodness-of-fit index; EP, number of parameters to be estimated.*

Inspection of maximum modification indices (Schumacker and Lomax, 2016) revealed that a correlation should be added between men’s and women’s narcissism. Despite the initial model yielding satisfactory fit, results of the final model indicated that, not only did it yield marginally better goodness-of fit when compared to the initial model (reduction in chi-square = 3.63; reduction in degrees of freedom = 1; resulting *p* < 0.10), but the correlation in particular was positive (*p* < 0.05). Addition of the correlation to the final model resulted in virtually no change in the magnitudes for the paths or correlations in the initial model (i.e., no change greater than 0.01 for paths; no change at all for correlations). Thus, whether the correlation between men’s and women’s narcissism is excluded or included, the conclusions to be drawn regarding exchanges of socioemotional rewards and costs between men and women are the same.

We note that, in the initial and final covariance structure analyses, the correlations between socioemotional rewards and costs were approximately −0.60 for each gender –significant, yet not as high as the correlations within the aforementioned confirmatory factor analyses (−0.80 for each gender) would have led us to expect. Furthermore, the magnitude of the reciprocal path coefficients linking (a) men’s and women’s socioemotional rewards as well as (b) men’s and women’s socioemotional costs did not appear to be adversely affected by potential multicollinearity between socioemotional rewards and costs within each gender (Cohen et al., 2003). Therefore, although we acknowledge concerns that regarding the interpretability of socioemotional rewards and costs as separable constructs in principle (for the Pilot Study and the Main Study), results of covariance structure analyses in the Main Study nonetheless affirmed the criterion-related validity of the separate subscales measuring socioemotional rewards and costs in practice (Nunnally and Bernstein, 1994).

Given that the chi-square for the final covariance structure model was below 2.00, it is statistically impossible for us to obtain further improvements in fit (whether significant or marginal) by adding any paths or correlations (Kline, 2016). Indeed, we are not aware of any theoretical *or* empirical rationale that would justify adding paths or correlations (Foa and Foa, 1974; Gaines, 1995; Gaines and Henderson, 2004). Therefore, we opted not to make any more changes to the model as displayed in Figure 1.

## General Discussion

We began with the assumption that the revised RBT (Gaines and Henderson, 2004) was best understood as a measure of affectionate and respectful behaviors, consistent with the resource exchange theory of Foa and Foa (1974). However, the results of our pilot and main studies led us to abandon that assumption. Clearly, the revised RBT should be understood as measuring *socioemotional rewards and costs*, consistent with the original interdependence theory (Thibaut and Kelley, 1959). Additionally, even though our discovery regarding the content of the revised RBT led us to hypothesize that narcissism would be reflected in patterns of reciprocity involving men’s and women’s socioemotional rewards and costs, the main study results were inconsistent with the hypothesis. By the same token, men’s and women’s narcissism were positively correlated. This was an unanticipated result that raises intriguing questions concerning the extent to which partners seek kindred spirits with regard to narcissism (see also Grosz et al., 2015). We concluded that, at best, we obtained partial support for our covariance structure model.

Why did rewards and costs (rather than affection and respect) emerge as the relevant behavioral dimensions in both studies? In general, exchange theories—including the resource exchange theory (Foa and Foa, 1974) and the original interdependence theory (Thibaut and Kelley, 1959)—implicitly or explicitly acknowledge the desirability of rewards for individuals in social and personal relationships (Dindia and Canary, 1993). However, interdependence theory is distinguished by its explicit framing of rewards and costs as major antecedents of relationship satisfaction (which, in turn, is a major antecedent of relationship commitment; Rusbult and Buunk, 1993). Perhaps rewards and costs were salient in the results of our exploratory and confirmatory factor analyses of the revised RBT (Gaines and Henderson, 2004), because rewards and costs are pivotal to individuals’ sense that the numerous wheels of relationship maintenance have been set into motion—a view that is consistent with findings from early tests of the *investment model* (Rusbult, 1980, 1983; Rusbult et al., 1986). In any event, our results concerning socioemotional rewards and costs complement previous findings (Carter et al., 2013) concerning the negative correlation between generic rewards and costs.

Why were men’s and women’s narcissism scores related positively but were unrelated to the bestowal of rewards or costs to one’s partner? One reason may be that our work depicted interactions between two persons who possess comparable levels of power and can be placed along a wide continuum from high to low narcissism, with the resulting two-person groups resembling “mutual admiration societies” (Grosz et al., 2015). Among our participants, similarity in levels of narcissism was evident; the matching process had no bearing upon their reciprocity of socioemotional rewards or costs (see also Lavner et al., 2016). Perhaps our results reflect dual processes at work: (1) Narcissism matches that involve individuals evaluating each other as suitable partners (possibly reflecting an ego-driven or self-enhancement motive; Sedikides and Gregg, 2008; Wallace, 2011); and (2) reward and cost matches that involves individuals calibrating their behaviors in a manner that allows them to maintain their relationships without placing themselves at a disadvantage with regard to dependence upon each other (perhaps reflecting a data-driven or accuracy motive; Rusbult and Van Lange, 2003).

### Strengths, Limitations, and Directions for Future Research

Our studies have certain strengths. For instance, to our knowledge, they are the first to progress beyond piecemeal *principal components analyses*—which are not theory-driven and do not yield estimates of latent variable scores, unlike maximum-likelihood versions of exploratory and confirmatory factor analyses (Tabachnick and Fidell, 2009)—in evaluating the psychometric properties of the revised RBT (Gaines and Henderson, 2004). Also, as far as we are aware, our main study is the first to test empirical links among men’s and women’s narcissism, socioemotional rewards, and socioemotional costs within a covariance structure model. Finally, the results of our main study concerning the impact of individuals’ socioemotional rewards and costs upon each other’s socioemotional rewards and costs *when covariance between individuals*’ *own socioemotional rewards and costs is taken into account* are fully consistent with an interdependence theory perspective (Rusbult and Van Lange, 2003).

Our studies also have certain shortcomings. For example, it is not clear whether the original RBT (Foa and Foa, 1974; Gaines, 1995) would yield the same factor pattern (i.e., socioeconomic rewards and costs, rather than affection-related and respect-related behaviors) that we obtained with the revised RBT (Gaines and Henderson, 2004), although the presence of “double-barreled” items in the original RBT (as we noted in the Method section of our pilot study) is problematic (Olson, 2008). Also, for our pilot study in particular, the sample size-to-*number of items* ratio (9.00) was somewhat smaller than the minimum desired level (i.e., 10.00 or higher; see Costello and Osborne, 2005, regarding sample size in exploratory factor analyses), though the main study yielded a sample size-to-*number of parameters* ratio (approximately 13.00) that was somewhat higher than the minimum desired level (i.e., 10.00 or higher; see Jackson, 2003, regarding sample size in confirmatory factor analyses). Lastly, our operationalization of individuals’ affection-giving, affection-denying, respect-giving, and respect-denying behaviors as words and deeds *to be reported by partners* might have impaired our ability to detect genuine effects of individuals’ narcissism *as reported by the individuals themselves* upon individuals’ socioemotional rewards and costs (for a broader discussion of difficulties in separating actor effects from perceiver effects within interdependence theory, see Kelley, 1997).

Regarding directions for future research, relationship scientists might wish to operationalize narcissism in terms of a circular or *circumplex model* (in the spirit of interpersonal circumplex theory; Wiggins et al., 1989), with lower-order aspects of narcissism arrayed in an equidistant manner around the psychological axes of *grandiose and vulnerable narcissism* (Miller et al., 2012). Such an innovation would help address criticism that the NPI (Raskin and Hall, 1979; Raskin and Terry, 1988), which we used in our main study, is limited to grandiose narcissism (Jauk and Kaufman, 2018). However, such a shift in methodology would require substantially larger sample sizes than we were able to obtain in the present studies (with minimum desired *n*’s ranging from 150 to more than 300 couples, depending on the complexity of the models to be tested; see Muthen and Muthen, 2002, concerning statistical power in confirmatory factor analyses).

### Implications for Therapy With Couples (and Individuals)

Despite our reconceptualization of individuals’ affection-giving, affection-denying, respect-giving, and respect-denying behaviors as socioemotional rewards and costs from the vantage point of interdependence theory (Thibaut and Kelley, 1959), we acknowledge that resource exchange theory (Foa and Foa, 1974) not only is compatible with interdependence theory (as articulated by Berg et al., 1993) but also may rival interdependence theory in terms of applicability to clinical practice as well as academic research (as contended by L’Abate and Harel, 1993). Furthermore, at the time that the pioneering books on interdependence theory and resource exchange theory were published, “narcissistic personality disorder” (denoting psychologically maladaptive forms of narcissism) had not received a formal designation within the American Psychiatric Association’s *Diagnostic and Statistical Manual of Mental Disorders*, or DSM (Millon, 1996), thus leading us to wonder whether results of the present studies would generalize from non-clinical to clinical populations. Although we did not have access to clinical samples, we are intrigued by the possibility that clinically narcissistic persons may instigate and reciprocate socioemotional costs toward partners (Sperry, 2003) in a confrontational manner (Black and Grant, 2014).

As Holmes (2004) observed, the social unit for interdependence theory has evolved from the *n*-person group (not necessarily defined by closeness; Thibaut and Kelley, 1959) to the two-person group (again, not necessarily defined by closeness; Kelley and Thibaut, 1978) to the relationship pair or dyad (by its nature, defined by closeness; Kelley, 1979). Results of the present studies indicate that (1) reciprocity of socioemotional rewards and (2) reciprocity of socioemotional costs are interrelated (yet separable) behavioral processes within heterosexual relationships (consistent with social exchange principles; Jacobson and Margolin, 1979). Although interdependence theorists (e.g., Kelley et al., 1983/2002) have acknowledged the widespread assessment of individual-level personality characteristics (including, but not limited to, quantitative and qualitative measures that reflect psychodynamic perspectives) within clinical practice, our results suggest that intervention may be most effective, if therapists target *couple*-level patterns of behavior (e.g., attempting to increase reciprocity of rewards and decrease reciprocity of costs, keeping in mind that it may be necessary to help some clients distinguish between short-term self-interest and long-term relationship maintenance; Kelley et al., 2003).

We note that individuals’ giving vs. denial of affection and respect *to themselves*—which we did not assess in the present studies—may be important data for therapists to collect as a means toward developing intervention strategies concerning clients’ *intra*personal, if not interpersonal, functioning (in line with social learning principles; Jacobson and Margolin, 1979). Also, given the over-emphasis on self-love and self-esteem that (stereo)typically characterizes persons whom therapists might diagnose as clinically narcissistic (Millon, 1996), our lack of covariance between individuals’ narcissism and their socioemotional behaviors toward partners should *not* be interpreted as evidence that psychodynamic personality constructs such as narcissism are irrelevant to social exchange processes as a whole (Kelley et al., 1983/2002). Nevertheless, such self-relevant behaviors on the part of clients might be especially important to the establishment, maintenance, and termination of *client-therapist relationships* (as distinct from the dynamics of clients’ relationships with significant others outside the clinical setting; Sullivan, 1956). In any event, a detailed examination of client-therapist relationships (including therapists’ behavior toward clients; Foa and Foa, 1974) is beyond the scope of the present paper.

### Concluding Thoughts

At the beginning of the present article, we alluded to Berscheid’s (1985) review concerning reinforcement-based theories of social psychology that have been applied to close relationship processes. We are aware that some relationship scientists (e.g., Clark and Lemay, 2010) might view our exchange-based view of relationship maintenance in heterosexual relationships as incompatible with the perspective (Clark and Mills, 1979) that ongoing relationships are subject to *communal* (rather than exchange) norms. However, we do *not* assume that exchange and communal norms are inherently opposed to each other (e.g., research on conflict resolution highlights the adaptiveness of partners’ refraining from engaging in *negative*, rather than positive, exchanges within close relationships; Fincham and Beach, 1999). Results of the present studies indicate that—as measured via the modified RBT (Gaines and Henderson, 2004; repurposed from Foa and Foa, 1974)—men’s and women’s exchanges involving socioeconomic rewards and costs constitute separate, yet related, relationship processes. In conclusion, we hope that the present article will encourage relationship scientists to (re)consider the possibility that certain aspects of social exchange can promote two-person group dynamics after all.

## Data Availability Statement

The raw data supporting the conclusions of this article will be made available by the authors, without undue reservation.

## Ethics Statement

The studies involving human participants were reviewed and approved by the Psychology Ethics Committee, Brunel University London. The patients/participants provided their written informed consent to participate in this study.

## Author Contributions

SG wrote the initial version of the manuscript. CS co-wrote final versions of the manuscript (with SG). Both authors contributed to the article and approved the submitted version.

## Conflict of Interest

The authors declare that the research was conducted in the absence of any commercial or financial relationships that could be construed as a potential conflict of interest.

## Publisher’s Note

All claims expressed in this article are solely those of the authors and do not necessarily represent those of their affiliated organizations, or those of the publisher, the editors and the reviewers. Any product that may be evaluated in this article, or claim that may be made by its manufacturer, is not guaranteed or endorsed by the publisher.
